# Supplementary material for: Five-year follow-up of fundus autofluorescence and retinal sensitivity in the fellow eye in exudative age-related macular degeneration in Japan
Source: PLoS One. 2020 Mar 6;15(3):e0229694. doi: 10.1371/journal.pone.0229694 (PMC7059919; doi:10.1371/journal.pone.0229694)
Supplement: Attachment — Submitted filename: review of PONE-D-19-28095.pdf [file pone.0229694.s001.pdf]

General comments

The authors report on a comparison between microperimetry and FAF patterns in a cohort of patients followed longitudinally for five years. Several are lost to follow-up or convert to CNV in the fellow eye, leaving 27 total eyes that were followed fully for five years. The authors show that test patches 'close' to abnormal areas seen on FAF decrease over three years. The authors show that one FAF pattern 'speckled' significantly decreased between both 3 and 5 years from baseline. Unfortunately, they only had one participant that exhibited this pattern, making it difficult to make generalizable conclusions based on a single observation. Another major drawback is that the authors only looked at FAF and did not assess how other pathology like large drusen at the test patch locations could have altered sensitivity in those areas, especially as drusen are dynamic and could change over the five years. The manuscript could be improved by better weighting of this finding with respect to the rest of the results as it seems like there is too much emphasis placed on this point. It is also difficult to understand how the FAF image classification changed over time because the authors state that for 14/27 followed for five years that they changed over time but it doesn't mention how they changed from year to year – this is particularly confusing with respect to figure 1 as this figure implies that the pattern stayed the same over time. This point needs to be clarified. There are also several specific items that could be altered to improve the overall presentation of the work, described below.

Specific comments

Abstract, lines 60-62: The wording of the descriptions of the classification of test points is awkward, please consider rephrasing.

Abstract, lines 69-71: This is confusing as written. Please rephrase from "…lost statistical significance…" to make this clearer.

Abstract, lines 72-73: The emphasis here on the speckled pattern relies on an n=1, perhaps the authors can add some additional points to this conclusion statement to less heavily weight this finding based on a single eye.

Introduction, line 77: The phrasing "…in the elderly in developed countries." Suggests that AMD only exists in developed countries, please rephrase.

Introduction, line 78: Some classification systems designate AMD as being AMD only if it is in persons >55 years of age, the authors should state the AMD classification system they are using here (e.g. Beckman, AREDS, etc.).

Introduction, line 84: Again, state the scale you are using to define intermediate AMD, etc.

Introduction, line 84-87: This entire sentence needs to be re-written as it is difficult to follow. Certain classification systems outline clearly what constitutes early, intermediate and advanced AMD, the authors should pick one to use and then clearly state the differences between the different stages here.

Introduction, line 91: As written, this is awkwardly phrased, particularly "…overweight, and genetic factors are considerable."

Introduction, line 95: It is unclear what the authors are intending to state here as microperimetry give a functional change, not a 'fundus change'. Please rephrase or reword this sentence.

Introduction, lines 100-101: This sentence does not seem to add anything and can be omitted.

Methods, lines 169-170: Who did the image grading? Was it always the same person? What criterion did they use to demarcate the areas manually? Were comparisons made between graders. It would be important for you to include enough detail here for someone else to replicate this experiment.

Results, general: The order the results are presented in does not make sense. The discussion of the transition between patterns (lines 265-269) needs to be described in better detail and should come before the presentation of the (lines 232-239) of three- and five-year findings.

Results, line 192: Replace "…autofluorescent examination…" with "…FAF imaging…".

Results, line 198: This sentence appears on its own and should be a part of the preceding paragraph. It should be restated here that only 27 eyes were followed for 5 years – that is how you get 44% from just 12 eyes.

Results, line 199-201: This sentence also could be merged into the preceding paragraph – it doesn't need to be its own paragraph.

Results, lines 233-239: Are these results for participants patterns only at the last timepoint? How did they change from the first timepoint, was there a difference between those whose patterns changed and those that stayed the same?

Results, 266-269, 279-281: This is confusing and needs to be completely rewritten. The authors may need to make a table or something describing the different transitions between different patterns. It cannot be understood fully from this prose how the patterns of 66 eyes changed over 5 years.

Discussion, line 292: The word "also" can be omitted from this sentence.

Discussion, lines 299-300: What is meant by the "low-fluorescent portion"? I think we need to see these images.

Discussion, lines 300-302: Do the authors mean progress to GA? They all have dry AMD so this does not make sense.

Discussion, line 304: It seems like the authors have the data to answer this question. Are the test patches that were 'close' now 'within' abnormal areas at year 3 and is this why they have the same sensitivity as 'within' patches from 3-5 years?

Discussion, line 311: Do the authors mean that it is a risk factor for AMD progression?

Discussion, line 313: What is meant by '…consistent with pigmentation…"?

Discussion, line 316: We need more information on what the patterns transitioned to so that this point can be better understood. Did the lace pattern transition to a less altered pattern?

Discussion, line 317: Do the authors mean "areas" here rather than "lesions"?

Discussion, line 318 & 319: Do the authors mean "test patches" here rather than "lesions"?

Discussion, line 325: The authors should state '…"Close" test points…" rather that '…"Close" patterns…'

Conclusions, general: This section needs to be completely rewritten, there is too much emphasis on the speckled pattern finding and there are some points that don't make sense (see specific comment below).

Conclusions, line 330: These patients all have dry AMD – see comment on this above.

Figure 1: Are these results for subjects whose patterns were always consistently the same at each timepoint? It is unclear what the number of subjects is in each pattern at each timepoint – maybe that could be included? Why do we have only 3 and 5 year timepoints shown here but we see sensitivity at a more granular level compared in figure 2?

Figure 2: The figure caption needs to be improved so it is clearer what is being tested for significance here at each timepoint (or if it is being tested to the first timepoint?). The (*) is compared to baseline timepoint but the (#) is compared to close at the same timepoint or at baseline? I think that a bar chart with lines showing comparisons may be needed to show exactly what was tested here. I think it would be useful for the authors to state whether the 'close' points at baseline would be graded as 'within' at the year 3 timepoint – it seems to be what is implied here. Are these test patches just now within abnormal areas or are they still 'close'?
