# Supplementary material for: Five-year follow-up of fundus autofluorescence and retinal sensitivity in the fellow eye in exudative age-related macular degeneration in Japan
Source: PLoS One. 2020 Mar 6;15(3):e0229694. doi: 10.1371/journal.pone.0229694 (PMC7059919; doi:10.1371/journal.pone.0229694)
Supplement: Attachment — Submitted filename: Responses to reviewers 20200123.docx [file pone.0229694.s002.docx]

We appreciate the comments from all reviewers. We reanalyzed our results to answer the questions from all reviewers. Therefore, we added one author (Takeshi Nishiyama) who analyzed and evaluated the results. We re-wrote the title and introduction for better understanding this manuscript.

NOTE: The order of results, references and figures have been changed.

**Response to the reviewer #1: We appreciate the comments of the reviewer. Specific concerns of the reviewer are addressed below:**

Reviewer #1:

1. P9 line 137: What do you mean “hyper- or hypofluorescence on FAF imaging in the fellow eye”? The follow eye has hypofluorescence but not (dry) AMD? Or it may has (dry) AMD also? If both eye has been diagnosed to have AMD, which eye would be considered as fellow eye? Although it has been stated in the previous study in the series, it is still worth a brief description in this paper.

→We rewrote this paragraph according to the reviewer’s suggestion.

**(Materials and Methods, Study Patients and Protocol section, 1^st^ paragraph, lines 138-151)**

2. Figure 1: standard deviation bar is missing. Also mark how many eyes and percentage of the 27 included for each pattern.

→ We added SE bar instead of SD bar in Fig2 (previous Fig1), because SD bars were beyond the figure frame. The percentage of FAF patterns (n=27) is different between baseline and the 5th year. Therefore, we created new Fig1 to show the percentage and the number of the eye in each FAF pattern. We modified previous Fig1 as Fig2 and added Year-2 and Year-4.

**(Results, Visual acuity and retinal sensitivity section, Fig2)**

3. Figure 2: mark how many spots and the percentage of total analyzed spots for distant, close and within.

→Since we created new Fig1, previous Fig2 is shown as Fig3 in our manuscript. We analyzed 1,215 points in total. To investigate the association between the retinal sensitivity and distance from the abnormal FAF, we changed the way of analysis using hierarchical linear mixed-effect models with the distance, time and time squared from baseline (months), and angle (degrees) as fixed effects. We found that the test patches which were “Close” group could turn into “Within” group during the 5-year. Therefore, we added new Table4 with Fig3 (previous Fig2). We deleted some descriptions from previous Fig2 because of changing the way of analysis.

**(Results, Retinal sensitivity and the distance section, Fig3 and Table 4)**

4. Sadly the only FAF pattern (speckled) shows worst progression and no pattern change has only 1 eye under study. Though the study cannot be re-done, yet the conclusion may not be convincing based on this very small sample size.

→We agree and have changed the conclusion. We deleted “The mean retinal sensitivity of the speckled FAF pattern and “Close” points tend to deteriorate after 3 years.” from the conclusion. We changed the conclusion in both abstract and manuscript.

**(Both conclusions in abstract and discussion)**

5. P19 Line293: In terms of the pattern change happened in this study during the 5 year, there is a brief description in the discussion but including a graph could be more helpful. How’s your pattern change trends compared to other studies?

→Thank you for your helpful comment. We created new figure as Fig1 for better understanding. Our pattern change was partially similar to other report. Bingöl Kızıltunç reported that focal increase pattern changed to patchy pattern in one eye (12.5%). In our study, focal increase pattern changed to patchy pattern in one eye (7.7%). We created the new table3 regarding pattern alterations observed during follow-up. Therefore, we added some description in Discussion, paragraph 2.

**(Results, Figure 1 and Table 3. Discussion, paragraph 3, lines 347-349)**

6. P20 Line303 How would you explain the “Close” pattern the sensitivity slightly “recovers” between 3 and 5 years? Worth more discussion.

→We found the retinal sensitivity between 3 and 5 years had no significant recovery. Although the retinal sensitivity between 3 and 5 years was low significantly compared to baseline, the retinal sensitivity at the 5^th^ year was not significant compared to the 3rd year.

7. P20 Line316

The reversible sensitivity also appears in lace-like pattern, what do you (and others) say about it?

→Thank you for your helpful comment. We discussed that in Discussion section.

**(Discussion, paragraph 2, lines 339-341)**

**Response to reviewer #2:**

**We appreciate the comments of the reviewer. Specific concerns of the reviewer are addressed below:**

This prospective, observational study assessed the 5-year changes of FAF and retinal sensitivity evaluated by microperimetry in the fellow eyes of Japanese patients with unilateral nAMD. I have the following questions:

1. Line 205-210, in this paragraph the authors described the data of the 14 eyes which progressed to nAMD (6 within Year 1 and 8 between Year 2 to Year 5), but in Table 2 the authors listed only the data of the 8 eyes progressed between Year 2 to Year 5, which made some data appear to be unmatched and confusing. Please clarify this.

→Thank you for your helpful comments. We rewrote the description in the 1^st^ paragraph. **(Results, Patient characteristics section, paragraph 1 and Table 1)**

2. Have the authors tried to compare the demographics, the FAF patterns, mean retinal sensitivity, as well as the % of “within”, “close”, and “distant” spots between the 8 eyes progressed to nAMD between 2-5 years and the other 6 eyes progressed within 1 year?

→ Thank you for your comments. The points you suggested are very important points. We would like to study what you pointed out in the future study. In this study, we focused on only 27 eyes without exudative change.

3. The exclusion criteria based on refractive error was < -8D, which was looser than most studies appoint -6D as the diagnostic criteria of high myopia. Previous studies showed macular sensitivity decreases with spherical equivalent. Given that East Asian countries have more high myopic patients than anywhere else over the world, I have a concern that if too many subjects in this study had relatively high myopia (between -6D to -8D), the retinal sensitivity results could be biased. Please comment on this point and if possible, exclude those with relatively high myopia.

→The points raised by the reviewer are very important points. We started this study by this protocol thorough the IRB. Therefore, we would like to exclude high myopia in the future study.

4. Line 218-222, is there any statistical significance between the baseline mean sensitivity of the 27 eyes without progression and the 14 eyes with progression, and between the baseline and Year 5 mean sensitivity of the 27 eyes? What’s the sensitivity of the 14 eyes at the endpoint? Or the last measurement before those eyes became wet?

→The points the reviewer suggested are very important points. In this study, we focused on only 27 eyes without exudative change. We would like to analyze the points raised by reviewer in the future study.

5. Line 238-239, “…deteriorated after 3 years compared to the other patterns (p < 0.001). *P < 0.05 compared to baseline, paired t-test.” Can the authors clarify what’s the statistical method used and how the comparisons were made here? Is it ever possible to use paired t-test to do this comparison (speckled VS other patterns)? Similarly, in Line 246-247, the method for conducting the statistical analysis should also be clearly stated in the Methods Section.

→Thank you for your helpful comments. We used paired t-test to compare the retinal sensitivity at each year compared to baseline. As the reviewer stated, we found that it was impossible to use paired t-test to compare other groups. Therefore, we deleted some description from Fig2 (previous Fig1). We rewrote Statistical analysis section in Materials and Methods. Note that the order of figure has changed.

**(Materials and Methods, Statistical analysis section)**

6. With the disease progression during the 5 years, I think it is likely that the prior “distant” point may later turn “close” or “within”, and the prior “close” point may later become “within”. If such alteration happened, how were the points classified? In Figure 2, the authors mentioned that paired t test was used. Does it mean that even a point has altered over time, it still belongs to the initial group?

→As you suggested, the prior “distant” point may later turn “close” or “within”, and the prior “close” point may later become “within”. Fig3 (previous Fig2) shows how the mean retinal sensitivity of test patches of each group at the baseline changed during the 5-year. In other words, test patches in the "Close" group at baseline are not necessarily the same "Close" group at the 5th year. We reanalyzed our results according to your suggestion. We wrote new methods to analyze these data in “Material and Methods section”. To investigate the association between the retinal sensitivity and distance from the abnormal FAF, hierarchical linear mixed-effect models were used with the distance, time and time squared from baseline (months), and angle (degrees) as fixed effects. Note that the order of figure has changed in manuscript.

**(Materials and Methods, Image analysis section, lines 179-186, and Statistical analysis section)**

7. Is it possible to divide each group (within, close, and distant) into 2 subgroups: progressive and non-progressive, and compare their sensitivity?

→　The points raised by the reviewer are very important points. We would like to investigate the points raised by the reviewer in the future study.

8. Line 252-255, unfortunately, the statistical methods were inappropriate no matter whether it was paired or unpaired t test, as the authors described in the Methods Section. When analyzing the 1215 points obtained from 27 eyes during multiple follow-ups, the data was not independent (multiple points from one eye) and repeated measurements were taken. Therefore, a mixed-effects model or a generalized estimating equation should be used. Also, the deterioration rate of the retinal sensitivity can be modeled by this means.

→Thank you for your helpful comments. According to your suggestion, we changed the method to analysis. We used hierarchical linear mixed-effect models. Therefore, we added new description and table for better understanding.

**(Materials and Methods, Statistical analysis section, and Results, Retinal sensitivity and the distance section)**

9. The test statistics (e.g., F, t) should be shown before the P value.

→Since we changed the way of analysis, we changed the description as shown new Table 4.

**(Results section, Table 4)**

10. Line 266-269, please clearly describe how many cases have changed from one pattern to another, with more detailed demographic features and the converted time of those eyes. A table is preferred.

→Since we were pointed out the similar suggestion from other reviewers, we added new Figure (as *fig*1) in Results section.

**(Results section, Fig1)**

11. The authors stated that the lace-like FAF pattern may be associated with retinal sensitivity reservation over time. However, there was only one such case involved in the study, and the slight increase of retinal sensitivity at Year 3 can also be attributed to the learning effect of the patient. More careful investigations should be conducted before this conclusion is made.

→Thank you for your helpful comment. Since we were pointed out the similar suggestion from other reviewers, we added new Figure (as *fig*1) in Results section and discussed the points in Discussion section as follows.

**(Results, Fig1 and Discussion, paragraph 2)**

12. Figure 1: Each line on this grey scale figure is very difficult to distinguish. Perhaps the authors may want to use a color figure for better illustration. Also, please add tick marks of each axis. If possible, data obtained at Year 2 & Year 4 could also be added.

→Thank you for the comment. We changed the Fig2 (previous Fig1) as a color figure according to the reviewer’s suggestion. We added tick marks of each axis and added Year 2 and Year 4 data in Fig2 (previous Fig1). Note that the order of figure has changed in manuscript.

**(Results, Fig2)**

13. As a psychophysics test, the results of microperimetry may be significantly affected by the examinees’ cognitive status, which is highly associated with older age and systemic health. It is not surprising that some older subjects get weaker and less cognitive during the 5-year follow-up period even though they are perfectly healthy at the time of enrollment. That’s why an age-matched control group is necessary. The lack of a control group should be discussed as a limitation of this study.

→ Thank you for your helpful comments. We added discussion as limitation as follows.

“Some older subjects may get weaker and less cognitive during the 5-year follow-up period even though they are perfectly healthy at the time of enrollment. An age-matched control group is necessary in the future study.”

**(Discussion section, paragraph 5, lines 362-365)**

14. Minor comments:

• Careful language polish may be needed. For example, in multiple places

(e.g., Line 190, Line 233, Line 234 …) “for” 5 years should be replaced by “during the”

→We changed many places from “for” 5 years to “during the”. Line 230 “up to the 5-year follow-up” should better be replaced by “during the 5 years”

**(Lines 70, 71, 76, 152, 190, 217 238, 245, 260-261, 272, 282, 318, 332 and 356)**

Line 236-237, “after 5 years” could be “The speckled FAF pattern had the lowest retinal sensitivity compared to the baseline at Year 5/at the 5th year”.

→We changed the portion as follows: The speckled FAF pattern had the lowest retinal sensitivity compared to the baseline at the 5th year.

**(Results, lines 266-267)**

Line 245, “at 5 years” should be “at the 5th year”.

→We changed the portion in Results section.

**(Results, line 275)**

Line 246, “after 3 years” should be “after the 3rd year”. The expression of “The speckled FAF pattern at 5 years was the least sensitive” should be “The speckled FAF pattern at the 5th years had the lowest mean retinal sensitivity”.

→We changed that part according to the reviewer’s suggestion. NOTE: The order of Results has been changed

**(Results, lines 275-276 and 285-286)**

Line 252, “more” should be “better”. There are more similar errors, the authors may need to carefully check it thoroughly.

→Thank you for your helpful comments. Since we changed the way of analysis using hierarchical linear mixed-effect models with the distance, time and time squared from baseline (months), and angle (degrees) as fixed effects, we deleted previous description.

• Caption of Figure 2: there is no data are expressed as mean ± standard error, in the figure.

→Previous Fig2 is now Fig3. We added the new analysis results as Table 4 with new Fig3 and changed the description.

**(Results, Fig3 and Table 4)**

• Line 198-199: need reformatting.

→We merged into the first paragraph of the results as follows. Twenty-seven eyes completed 5 years of follow-up with both microperimetry and FAF imaging (right eyes were 12 (44.4%) (Table 2).

**(Results, Patient characteristics, paragraph 2, lines 230-231)**

• Line 205-205: Polypoidal choroidal vasculopathy, wet AMD, and retinal angiomatous proliferation were detected in 4, 9, and 1 eyes, respectively (Table 1). But Table 1 didn’t contain such information.

→Thank you for your helpful comments. We rewrote the description in the 1^st^ paragraph for better understanding.

**(Results, Patient characteristics section, 1st paragraph)**

• Line 209: lost should be loss.

→Thank you for your helpful comments. We rewrote that portion in red letters.

**(Results, patient characteristics, 2nd paragraph, line 227)**

• Line 209-210, “The number of patients lost to follow-up was 2, 5, 6, 3, and 3 at the 1-, 2-, 3-, 4-, and 5-year follow-ups”, please move this sentence to Line 191, after “Of the remaining 191 eyes”, in order to minimize any confusion.

→Thank you for your helpful comments. We moved the portion that the reviewer suggested.

**(Results, patient characteristics, 2nd paragraph, lines 227-228)**

• Line 310, “after” 5 years may be inaccurate.

→Thank you for your comments. We rewrote the portion as follows.

“The remaining patchy FAF pattern (6 eyes (22.2%) at baseline) showed no significant change in retinal sensitivity until the 3rd year but showed a significant decrease in retinal sensitivity at the 3rd, 4th and 5th year compared to baseline (10 eyes (37.0%) at the 5th year) (Fig 2).”

**(Discussion section, paragraph 2, lines 332-336)**

**Response to reviewer #3:**

**We appreciate the comments of the reviewer. Specific concerns of the reviewer are addressed below:**

Reviewer #3: General comments

The authors report on a comparison between microperimetry and FAF patterns in a cohort of patients followed longitudinally for five years. Several are lost to follow-up or convert to CNV in the fellow eye, leaving 27 total eyes that were followed fully for five years. The authors show that test patches ‘close’ to abnormal areas seen on FAF decrease over three years. The authors show that one FAF pattern ‘speckled’ significantly decreased between both 3 and 5 years from baseline. Unfortunately, they only had one participant that exhibited this pattern, making it difficult to make generalizable conclusions based on a single observation. Another major drawback is that the authors only looked at FAF and did not assess how other pathology like large drusen at the test patch locations could have altered sensitivity in those areas, especially as drusen are dynamic and could change over the five years. The manuscript could be improved by better weighting of this finding with respect to the rest of the results as it seems like there is too much emphasis placed on this point. It is also difficult to understand how the FAF image classification changed over time because the authors state that for 14/27 followed for five years that they changed over time but it doesn’t mention how they changed from year to year – this is particularly confusing with respect to figure 1 as this figure implies that the pattern stayed the same over time. This point needs to be clarified. There are also several specific items that could be altered to improve the overall presentation of the work, described below.

Specific comments

1．Abstract, lines 60-62: The wording of the descriptions of the classification of test points is awkward, please consider rephrasing.

→Thank you for your comment. We changed the description in the abstract as the reviewer suggested.

**(Abstract, lines 60-63)**

2．Abstract, lines 69-71: This is confusing as written. Please rephrase from “…lost statistical significance…” to make this clearer.

→We reanalyzed our results, and as a result, our method and results had been changed in abstract (in red letters).

**(Materials and Methods and Results)**

3．Abstract, lines 72-73: The emphasis here on the speckled pattern relies on an n=1, perhaps the authors can add some additional points to this conclusion statement to less heavily weight this finding based on a single eye.

→We agree and have changed the conclusion as follows.

“FAF patterns can change about half during the 5 years and the retinal sensitivity near abnormal FAF tends to deteriorate after the third year.”

**(Abstract, lines 76-77)**

4．Introduction, line 77: The phrasing “…in the elderly in developed countries.” Suggests that AMD only exists in developed countries, please rephrase.

→As the reviewer suggested, AMD does not exist necessarily in developed countries. Therefore, we deleted “in developed countries” from the sentence.

**(Introduction, line 80)**

5．Introduction, line 78: Some classification systems designate AMD as being AMD only if it is in persons >55 years of age, the authors should state the AMD classification system they are using here (e.g. Beckman, AREDS, etc.).

→We used the AMD classification of AREDS. Therefore, we re-wrote this entire paragraph.

**(Introduction, paragraph 1)**

6．Introduction, line 84: Again, state the scale you are using to define intermediate AMD, etc.

→We re-wrote 1st and 2nd paragraphs.

**(Introduction, paragraph 1)**

7．Introduction, line 84-87: This entire sentence needs to be re-written as it is difficult to follow. Certain classification systems outline clearly what constitutes early, intermediate and advanced AMD, the authors should pick one to use and then clearly state the differences between the different stages here.

→We re-wrote this entire sentence in paragraph 1 according to AREDS report:

**(Introduction, paragraph 1)**

8．Introduction, line 91: As written, this is awkwardly phrased, particularly “…overweight, and genetic factors are considerable.”

→We agree and have changed the description.

**(Introduction, lines 95-97)**

9．Introduction, line 95: It is unclear what the authors are intending to state here as microperimetry give a functional change, not a ‘fundus change’. Please rephrase or reword this sentence.

→We rewrote as follows: Microperimetry is a useful tool to detect a slight functional change and fundus autofluorescence (FAF) is a useful tool to detect a slight fundus change non-invasively.

**(Introduction, lines 98-100)**

10．Introduction, lines 100-101: This sentence does not seem to add anything and can be omitted.

→We agree and have deleted the description, as suggested.

11．Methods, lines 169-170:

Who did the image grading?

→Four authors (ST, MS, YO, and TY) graded the images into the following 8 patterns: minimal change, focal increase, focal plaque-like, patchy, linear, lace-like, reticular, and speckled.

**(Materials and Methods, Image analysis section, lines 168-170)**

Was it always the same person?

→Yes, it was.

What criterion did they use to demarcate the areas manually?

→The border of abnormal area was measured at the discretion of the individual. The decision of the area on FAF was made by one retina expert (TY) for all cases.

**(Materials and Methods, Image analysis section, lines 173-174)**

Were comparisons made between graders.

→The comparison were not made between graders.

12．Results, general: The order the results are presented in does not make sense. The discussion of the transition between patterns (lines 265-269) needs to be described in better detail and should come before the presentation of the (lines 232-239) of three- and five-year findings.

→As the reviewer suggested, we changed the order of the results before Fig2 (previous Fig1).

**(Results, Transition to other FAF patterns section and Table 3 )**

13．Results, line 192: Replace “…autofluorescent examination…” with “…FAF imaging…”.

→As you suggested, we replaced that part.

**(Results, Patient characteristics, paragraph 2, line 231)**

14．Results, line 198: This sentence appears on its own and should be a part of the preceding paragraph. It should be restated here that only 27 eyes were followed for 5 years – that is how you get 44% from just 12 eyes.

→　We merged into the second paragraph of the results as follows.

**(Results, Patient characteristics, paragraph 2, lines 230-231)**

15．Results, line 199-201: This sentence also could be merged into the preceding paragraph – it doesn’t need to be its own paragraph.

→We merged into the preceding paragraph.

**(Results, Patient characteristics, paragraph 1, lines 219-223)**

16．Results, lines 233-239: Are these results for participants patterns only at the last timepoint? How did they change from the first timepoint, was there a difference between those whose patterns changed and those that stayed the same?

→Thank you for your questions. The percentage of FAF patterns (n=27) is different between baseline and the 5th year. Therefore, we created new Fig1 to show the percentage and the number of the eye in each FAF pattern. We modified previous Fig1 as Fig2 and added Year-2 and Year-4. We discussed this question in Discussion section,

**(Results Fig1, Fig2 and Fig3, and Discussion, paragraph 2.**

17．Results, 266-269, 279-281: This is confusing and needs to be completely rewritten. The authors may need to make a table or something describing the different transitions between different patterns. It cannot be understood fully from this prose how the patterns of 66 eyes changed over 5 years.

→　According to the reviewer’s suggestion, we completely rewrote those parts as paragraph 1 and we added new Fig1 and Table3. NOTE: The order of Results and the order of figures have been changed.

**(Results section, Patient characteristics, paragraph 1, Fig1, and Table 3).**

18．Discussion, line 292: The word “also” can be omitted from this sentence.

→Thank you for your comments. As the reviewer suggested, we deleted the word “also”.

**(Discussion, lines 330)**

19．Discussion, lines 299-300: What is meant by the “low-fluorescent portion”? I think we need to see these images.

→Thank you for your suggestion. Hypo-fluorescent portion is shown in Fig4 in part.

**(Results, Fig4 and Fig4’s description)**

20. Discussion, lines 300-302: Do the authors mean progress to GA? They all have dry AMD so this does not make sense.

→Thank you for your comment. As the reviewer suggested, these sentences did not make sense. Therefore, we delete these sentences.

21．Discussion, line 304: It seems like the authors have the data to answer this question. Are the test patches that were ‘close’ now ‘within’ abnormal areas at year 3 and is this why they have the same sensitivity as ‘within’ patches from 3-5 years?

→　Thank you for your comments. Other reviewer suggested this point similarly. Therefore, we added some descriptions in Discussion.

**(Discussion, paragraph 4)**

22．Discussion, line 311: Do the authors mean that it is a risk factor for AMD progression?

→Thank you for your comments. In this study, the number of patients’ loss to follow-up was big. There were 10 eyes with a patchy pattern that did not actually cause AMD. Therefore, we deleted these descriptions.

23．Discussion, line 313: What is meant by ‘…consistent with pigmentation…”?

→Thank you for your comments. We added some description in Discussion as follows.

“It is possible that the lace-like pattern may be associated with RPE hyperplasia or other transient stressed condition. Because the lace-like pattern was not co-localized with drusen.”

**(Discussion section, paragraph 2, lines 339-341)**

24．Discussion, line 316: We need more information on what the patterns transitioned to so that this point can be better understood. Did the lace pattern transition to a less altered pattern?

→Thank you for your comment. Since we were pointed out the similar suggestion from other reviewers, we created new Figure as Fig1. We also discussed this point in Discussion.

**(Results, Fig 1 and Discussion, paragraph 2)**

25．Discussion, line 317: Do the authors mean “areas” here rather than “lesions”?

→Thank you for your comments. As the reviewer suggested, we changed the word as areas.

**(Discussion, paragraph 4, line 350)**

26．Discussion, line 318 & 319: Do the authors mean “test patches” here rather than “lesions”?

→Thank you for your comments. As the reviewer suggested, we changed the word from “lesions” to “test patches”.

**(Discussion, paragraph 4, lines 351 and 355)**

27．Discussion, line 325: The authors should state ‘…”Close” test points…” rather that ‘…”Close” patterns…’

→Thank you for your comments. We deleted this description from our manuscript because we changed the conclusion.

28．Conclusions, general: This section needs to be completely rewritten, there is too much emphasis on the speckled pattern finding and there are some points that don’t make sense (see specific comment below).

→As you suggested, we re-wrote completely our conclusion.

**(Conclusions, lines 367-373)**

29．Conclusions, line 330: These patients all have dry AMD – see comment on this above.

→Thank you for your helpful comments. According to the reviewer’s suggestion, we completely rewrote conclusion.

**(Conclusions, lines 367-373)**

30. Figure 1: Are these results for subjects whose patterns were always consistently the same at each timepoint? It is unclear what the number of subjects is in each pattern at each timepoint – maybe that could be included?

→Thank you for your helpful comments. These results for subjects whose patterns were not always consistently the same at each timepoint. Other reviewers suggested similar points. For better understanding, we created new Figure as Fig1 to show the percentage and the number of the eye in each FAF pattern. in Results section.

**(Results, Fig1)**

Why do we have only 3 and 5 year timepoints shown here but we see sensitivity at a more granular level compared in figure 2?

→We modified previous Fig1 as Fig2 and added Year-2 and Year-4. We added new Table 4 to explain Fig3 (previous Fig2).

**(Results, Fig2, Fig3 and Table4)**

31．Figure 2: The figure caption needs to be improved so it is clearer what is being tested for significance here at each timepoint (or if it is being tested to the first timepoint?). The (*) is compared to baseline timepoint but the (#) is compared to close at the same timepoint or at baseline? I think that a bar chart with lines showing comparisons may be needed to show exactly what was tested here. I think it would be useful for the authors to state whether the ‘close’ points at baseline would be graded as ‘within’ at the year 3 timepoint – it seems to be what is implied here. Are these test patches just now within abnormal areas or are they still ‘close’?

→Thank you for your comments. Since we reanalyzed our results, we deleted the (*) and the (#) from Fig3 (previous Fig2) and added new Table (Table 4) for better understanding. As the reviewer suggested, we improved figure caption. This figure shows how the mean retinal sensitivity of test patches of each group at the baseline changed during the 5-year. In other words, test patches in the "Close" group at baseline are not necessarily the same "Close" group at the fifth year.

**(Results, Fig3 caption and Table4)**
